# Supplementary material for: Extracellular Matrix Changes in Subcellular Brain Fractions and Cerebrospinal Fluid of Alzheimer’s Disease Patients
Source: Int J Mol Sci. 2023 Mar 14;24(6):5532. doi: 10.3390/ijms24065532 (PMC10058969; doi:10.3390/ijms24065532)
Supplement: Supplementary file 1 [file ijms-24-05532-s001.zip › Suppl Figure S1.pdf]

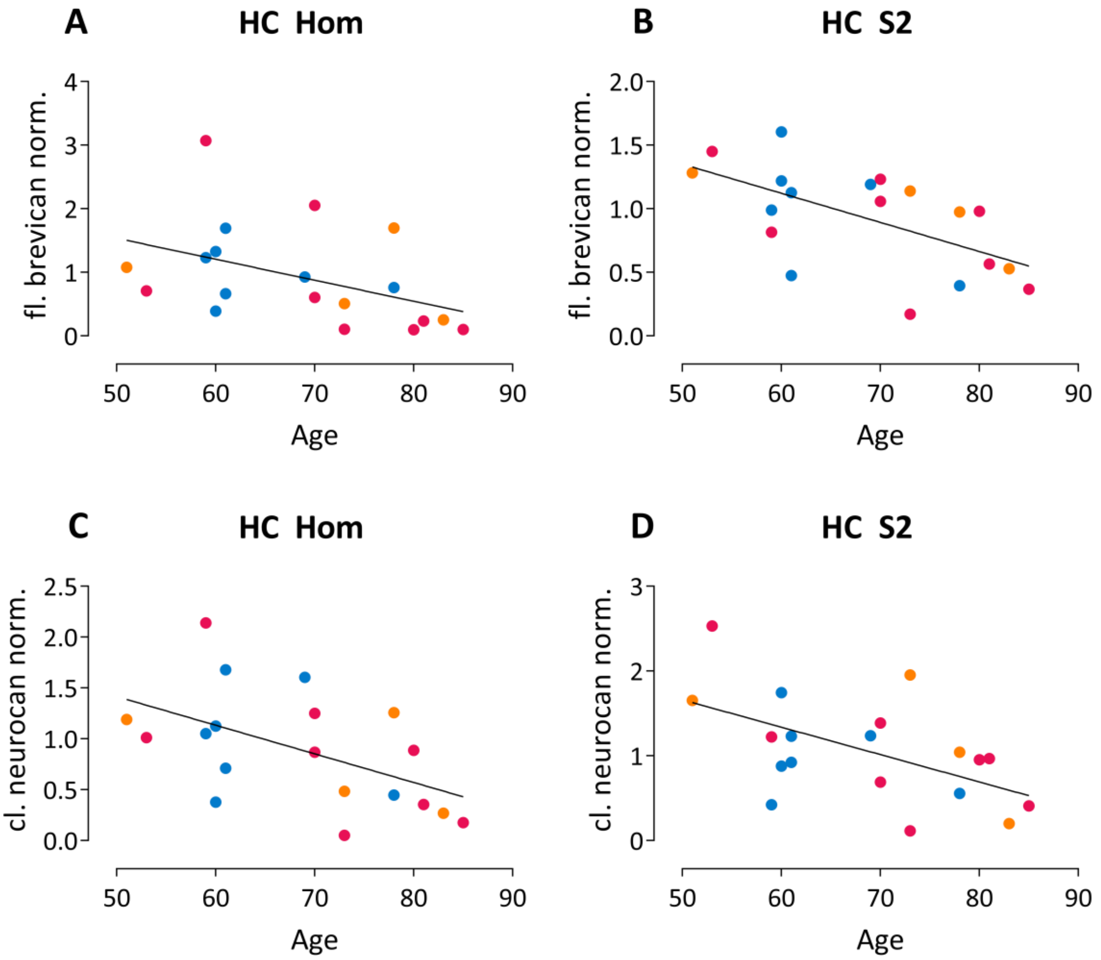

Figure S1: Spearman's partial correlations corrected for the variable diagnosis for full-length brevican in (A) hippocampal homogenate (ϱ = −0.528; $p = 0.024$) and (B) soluble fractions (ϱ = −0.621; $p = 0.006$), and also for the 130 kDa neurocan fragment in (C) homogenates (ϱ = −0.534; $p = 0.022$) and (D) S2 soluble fractions (ϱ = −0.501; $p = 0.034$). Color code for diagnostic groups is indicated.
